# Silent Tumefactive Demyelinating Lesions and Radiologically Isolated Syndrome

**DOI:** 10.1155/2018/8409247

**Published:** 2018-11-28

**Authors:** Ozgul Ekmekci, Cenk Eraslan

**Affiliations:** ^1^Ege University, Faculty of Medicine, Department of Neurology, Izmir, Turkey; ^2^Ege University, Faculty of Medicine, Department of Radiology, Izmir, Turkey

## Abstract

Demyelinating lesions larger than 2 cm in diameter, with or without edema, are known as tumefactive demyelinating lesions (TDLs). They constitute a rare inflammatory demyelinating disorder of the central nervous system. TDLs are typically characterized by headaches, cortical symptoms such as aphasia, hemiparesis, hemisensory deficits, seizures, and changes in consciousness. TDLs may occur in patients with or without an established diagnosis of MS or may occur as the initial demyelinating event. They may also be observed during follow-up in patients with MS, neuromyelitis optica, acute disseminated encephalomyelitis, or other autoimmune diseases. Differential diagnosis includes brain tumors, abscess, granulomatous diseases, and vasculitis. In some cases, it may be very difficult to distinguish TDLs from a tumor, such that biopsy might be needed. However, no cases of asymptomatic TDLs have yet been reported in the literature. Hence, in this report, we present a case involving an asymptomatic TDL detected incidentally during magnetic resonance imaging in an 18-year-old man. The patient did not develop any symptoms during the 1-year follow-up period. During follow-up, the patient was diagnosed with a radiologically isolated syndrome. TDLs have not previously been identified as radiologically isolated syndrome. Thus, reporting similar cases in the future will help in further understanding this phenomenon.

## 1. Introduction

A tumefactive demyelinating lesion (TDL) is an inflammatory demyelinating disorder of the central nervous system (CNS), which may occur in patients with or without an established diagnosis of multiple sclerosis (MS). TDLs are defined as tumor-like demyelinating lesions larger than 2 cm, with or without surrounding edema, mass effect, and ring enhancement. These lesions are often found in the supratentorial region, most commonly in the frontal and parietal lobes. Furthermore, basal ganglia, infratentorial and spinal cord lesions, as well as butterfly lesions involving the corpus callosum, can also occur. Tumefactive lesions typically affect white matter, but gray matter may also be affected [1]. TDLs can occur as single or multiple lesions and may appear simultaneously or sequentially [2]. Magnetic resonance imaging (MRI) findings of TDLs include mass effect, edema, T2 hypointense rim, T1 hypointensity, contrast enhancement with gadolinium (exhibiting homogenous, heterogeneous, nodular, punctate, or open/closed ring patterns), and peripheral restriction around the lesion on diffusion-weighted images (DWI). MR spectroscopy typically demonstrates an increased choline/N-acetyl-aspartate ratio, reduced N-acetyl-aspartate/creatine ratio, and increased choline/creatine ratio [1, 3, 4]. TDLs should be distinguished from granulomas, infections, vasculitis, and neoplasms (e.g., glial tumors and primary CNS lymphoma) [3]. Perfusion MRI techniques can be used to distinguish malignant demyelinating lesions from tumors. The mean relative cerebral blood volume in patients with TDLs is less than that of brain tumors. However, this technique cannot distinguish primary CNS lymphoma [3]. Furthermore, an increased choline/N-acetyl-aspartate ratio in MR spectroscopy is a common finding in tumors, as in TDLs [5].

When clinical and imaging features are typical, biopsy may be unnecessary [6]. Furthermore, TDLs exhibit high cellularity, which may lead to misdiagnosis as glioblastoma [1]. However, differential diagnosis may sometimes be difficult, and biopsy might be needed.

Clinical presentation of TDLs often differs from MS, depending on the lesion location and size. Headache, cognitive abnormalities, confusion, changes in consciousness, aphasia/dysphasia, apraxia, hemiparesis, hemisensory deficits, visual field deficits, and seizures can occur due to TDLs [1]. However, cases of asymptomatic TDLs have not yet been reported in the literature.

The term “radiologically isolated syndrome (RIS)” was first used by Okuda et al. [7, 8] to define individuals without overt clinical symptoms but with MRI finding suggestive of MS. Okuda et al. proposed diagnostic criteria for RIS in 2009 (Table 1) [8].

Okuda described CNS demyelination as a “silent thief” that disturbs neuronal and cellular functions slowly and only becomes apparent when a threshold is exceeded [7]. Several studies have shown that up to 30% of patients with RIS developed a demyelinating attack within 5 years and two-thirds progressed radiologically. Furthermore, approximately 10% of patients with RIS evolved to primary progressive MS [9]. Young age at onset (< 37 years), male gender, and presence of demyelinating lesions on the spinal cord were found as the most relevant predictors for the development of MS. Oligoclonal bands and an increased IgG index in CSF have been found as risk factors when combined with other paraclinical and radiological findings. Gadolinium-enhancing lesions have been identified as a risk factor in some studies but not in others. The presence of lesions on the spinal cord is considered as the strongest risk factor. Furthermore, it has been debated whether RIS is truly asymptomatic. Cognitive impairment was determined in 20–-30% of patients with RIS. Affected cognitive domains in RIS are similar to those in MS and include attention, information processing speed, memory, and executive functions. Psychiatric disorders such as depression, anxiety, and somatization are found more frequently in patients with RIS than healthy controls. Investigators have suggested that these disturbances are associated with white matter lesions and normal appearing white matter damage [10].

Herein, we have presented a case with RIS and asymptomatic TDLs and the associated findings during radiological follow-up of the affected patient.

## 2. Case

An 18-year-old man was referred to our outpatient clinic because of unique MRI findings. MRI was performed by another physician because the patient exhibited tremor in both hands, which began 1 year earlier. However, this tremor did not affect his quality of life. His father had also exhibited a similar tremor in both hands for many years. There was no history of previous health problems. The patient did not complain of headache, fever, arthritis, or skin rash. He had no behavioral, psychiatric, or cognitive complaints. There was no history of vaccination or infection history before the MRI was performed. Detailed neurological examination of the patient revealed only bilateral postural tremor in his hands. No aphasia, apraxia, cortical sensory disturbance, or visual field defect was detected upon examination.

**Figure 1:**
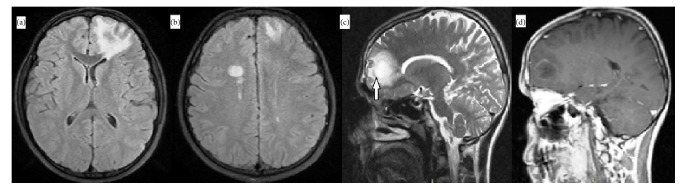
Initial imaging findings. (a) Axial fluid attenuation inversion recovery (FLAIR) image reveals a large hyperintense lesion in the left frontal lobe with surrounding vasogenic edema. (b) Axial FLAIR image shows hyperintense demyelinating lesions in bilateral subcortical white matter. (c) Sagittal T2-weighted image demonstrates T2 hypointense rim (arrow). (d) Sagittal T1-weighted postcontrast image shows ring enhancement.

MRI revealed a tumefactive edematous lesion in the left frontal area, which was hypointense on T1-weighted images, and hyperintense on T2-weighted and fluid attenuation inversion recovery (FLAIR) images. After gadolinium administration, T1-weighted images demonstrated ring enhancement. Hyperintense lesions were observed in the subcortical and deep white matter in the right hemisphere on T2-weighted and FLAIR images (Figure 1). Spinal MRI was normal.

Complete blood count, erythrocyte sedimentation rate, and levels of biochemical parameters, including glucose, urea, creatinine, lactate dehydrogenase, activities of alanine aminotransferase, and aspartate aminotransferase, were normal. Vasculitis screening tests for ANA, ANCA, anti-DNA, anti-Ro, and anti-La were negative. The anti-HIV 1/2 test was also negative. Cerebrospinal fluid (CSF) analysis revealed a normal cell count, as well as normal levels of protein and glucose; oligoclonal bands were observed in the CSF that were absent from in the serum. Anti-NMO and anti-MOG antibodies were negative. Thoracic computed tomography and abdominal ultrasonography were normal. Studies of visual evoked potential revealed prolonged P100 latency in the right eye. The optical coherence tomography was normal and the contrast sensitivity was also normal.

Neuropsychological examination was performed to determine the cognitive dysfunction. The Stroop test, PASAT-3 (Turkish), and BICAMS battery (symbol digit modality test, SDMT; California verbal learning test-II, CVLT-II; brief visuospatial memory test revised, BVMTR) were used to assess cognitive performance. His PASAT-3 score was 60/60. Stroop test, SDMT, CVLT-II, and BVMTR scores were within normal values.

A 10-Hz symmetric tremor was recorded with surface electromyography (EMG) from the forearm flexor and extensor muscles. Clinical and electromyographic features and positive family history supported an essential tremor diagnosis. The tremor was not associated with demyelinating lesions in MRI images and was therefore regarded as an essential tremor.

The patient was not treated and underwent radiological follow-up.

**Figure 2:**
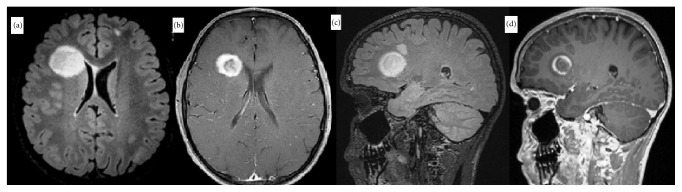
One month later. ((a),(c)) Improvement of the tumefactive lesion is visible in the left frontal lobe and a new tumefactive lesion has appeared in the right frontal white matter. ((b),(d)) T1-weighted postcontrast images show ring enhancement.

**Figure 3:**
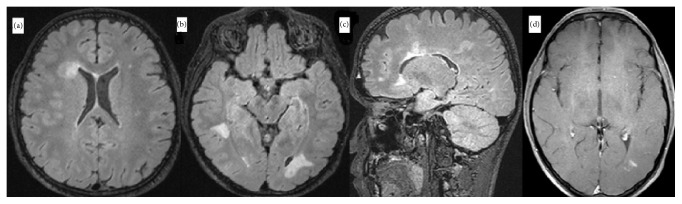
One year later. (a) Regression of tumefactive lesions is evident. (b) Axial fluid attenuation inversion recovery (FLAIR) image shows new hyperintense lesions in the bilateral parietooccipital white matter. (c) Sagittal FLAIR image demonstrates periventricular, cortical/juxtacortical demyelinating lesions. (d) T1-weighted postcontrast image shows contrast enhancement of the lesion in the left parietooccipital white matter.

Follow-up MRI performed one month later revealed an improvement of the tumefactive lesion in the left frontal area. However, a new tumefactive lesion with perifocal edema, which demonstrated ring-like gadolinium enhancement on T1-weighted images and peripheral restriction on DWI, was observed in the right frontal area (Figure 2). One year later, a follow-up brain MRI showed regression of the tumefactive lesion, as well as two new hyperintense lesions on T2-weighted and FLAIR images in the bilateral parietooccipital areas. After gadolinium administration, peripheral contrast enhancement was observed in the lesion on the left side (Figure 3). Spinal MRI was normal.

The patient was monitored without treatment. One year later, a follow-up was conducted, which revealed that he had not developed any obvious neurological symptoms. Written informed consent was obtained from the participant for the publication of this case report.

## 3. Discussion

TDLs in clinically isolated syndrome (CIS) and MS cases typically occur during the first demyelinating attack in most patients. However, these lesions can also occur during follow-ups in patients with known MS. TDLs associated with treatment with fingolimod and natalizumab treatment have been reported in patients with MS. TDLs can also occur during other inflammatory diseases, such as acute disseminated encephalomyelitis (ADEM), acute hemorrhagic leukoencephalopathy, NMO, and MS variants (e.g., Schilder's disease, Marburg's disease, and Balo's concentric sclerosis). TDLs have also been reported during viral infection (HIV), malignancy (renal cell carcinoma), autoimmune diseases (e.g., Sjögren's syndrome, Neuro-Behçet's disease, or lupus erythematosus) [6, 11, 12]. Differential diagnosis includes glial tumors, primary CNS lymphoma, metastasis, and brain abscess. Brain biopsy may be necessary in some cases. In the presented case, no vaccination or infection had occurred before the first MRI. There were no complaints or symptoms of systemic vasculitis, such as dry eyes, skin rash, skin ulcer, arthritis, or weight loss. Systemic vasculitis markers were negative. Thoracic and abdominal imaging, used to investigate malignancy, were normal. In the present case, the initial TDL regressed spontaneously over 1 month, but a new TDL developed in the contralateral white matter. Glial tumor and lymphoma were excluded because of these serial radiological features, as well as the presence of oligoclonal band positivity in the CSF. One year later, new MRI findings fulfilled the Barkhof criteria (DIS) for conversion to RIS. White matter lesions were not consistent with a vascular pattern, and there was no history of drug abuse, toxic exposure, or a medical condition. He was still asymptomatic and there were no impairments in social, occupational, or generalized functions. The final diagnosis was accepted as RIS.

In the present case, the essential tremor was not associated with TDLs, suggesting that the TDLs were asymptomatic and were solely detected incidentally on MRI images. Thus far, asymptomatic TDLs have not been reported in the literature, except in patients with known MS. The initial MRI findings of the patient in this report do not meet the criteria for RIS. Follow-up of this patient revealed that the asymptomatic TDLs progressed to a classical form of RIS. A new concept, "RIS-like TDLs" or “silent tumefactive demyelinating lesions” may be appropriate in this case. In the literature, it has been reported that 66-70% of patients with TDL as the initial demyelinating event had progressed to clinically definite MS [1, 13]. Our patient remains asymptomatic and has continued monitoring without treatment. This patient is in a high-risk group due to his younger age, male gender, gadolinium-enhancing lesions, and TDLs. Treatment of RIS with disease-modified therapy (DMT) is still controversial. A group of researchers recommends treatment for patients at high risk. However, it is not certain which criteria and markers should be used at the start and follow-up of the treatment [10]. Therefore we have not started treatment with DMT.

Conclusions from this case report include the following: (1) MRI activity is not always equivalent to clinical appearance. (2) We do not know the mechanisms that prevent symptoms despite the higher MR activity in this patient and other patients with RIS. (3) How we can classify silent demyelinating lesions that do not fulfill the Barkhof criteria for DIS. To our knowledge, this is the first report to describe asymptomatic TDLs. In terms of RIS and its diagnostic criteria, the specific characteristics of tumefactive lesions are not clear. Reporting similar cases in the future may clarify whether such demyelinating lesions can be included as RIS.

Tumefactive lesions may also be presented as RIS-like appearance. The contribution of silent tumefactive lesions to risk for development of MS in RIS-like appearance will be determined by long-term follow-up.

## Figures and Tables

**Table 1:** Proposed diagnostic criteria for radiologically isolated syndrome [8].

(A) The presence of incidentally identified CNS white matter anomalies meeting the following MRI criteria
(1) Ovoid, well-circumscribed and homogeneous foci with or without involvement of the corpus callosum
(2) T2 hyperintensities measuring >3 mm and fulfilling Barkhof criteria (at least three out of four) for dissemination in space
[(i) the presence of at least one gadolinium enhancing lesion or nine T2 hyperintense lesions; (ii) the presence of at least one infratentorial lesion; (iii) the presence of at least one juxtacortical lesion; and (iv) the presence of at least three periventricular lesions]
(3) CNS white matter anomalies not consistent with a vascular pattern
(B) No historical accounts of remitting clinical symptoms consistent with neurological dysfunction
(C) The MRI anomalies do not account for clinically apparent impairments in social, occupational or generalized areas of functioning
(D) The MRI anomalies are not due to the direct physiological effects of substances (recreational drug abuse, toxic exposure) or a medical condition
(E) Exclusion of individuals with MRI phenotypes suggestive of leukoaraiosis or extensive white matter pathology lacking involvement of the corpus callosum
(F) The CNS MRI anomalies are not better accounted for by another disease process
